# Bird-livestock interactions associated with increased cattle fecal shedding of ciprofloxacin-resistant *Escherichia coli* within feedlots in the United States

**DOI:** 10.1038/s41598-020-66782-4

**Published:** 2020-06-23

**Authors:** James C. Carlson, Jeffrey C. Chandler, Bledar Bisha, Jeffrey T. LeJeune, Thomas E. Wittum

**Affiliations:** 1 U.S. Department of Agriculture, Animal and Plant Health Inspection Service, Wildlife Services, National Wildlife Research Center, Fort Collins, CO USA; 2 Department of Animal Science, College of Agriculture and Natural Resources, University of Wyoming, Laramie, WY USA; 3 The Ohio State University, Wooster, OH USA; 4 Present Address: Food and Agriculture Organization of the United Nations (FAO), Viale delle Terme di Caracalla, 00153 Rome, Italy; 5 Department of Veterinary Preventive Medicine, College of Veterinary Medicine, The Ohio State University, Columbus, OH USA

**Keywords:** Antimicrobial resistance, Invasive species, Bacteriology

## Abstract

This research study was conducted to determine if bird depredation in feedlots is associated with the prevalence of ciprofloxacin-resistant *Escherichia coli* in cattle and to determine if removal of invasive bird species could be an effective management strategy to help reduce ciprofloxacin-resistant *E. coli* in cattle within the United States. European starlings (*Sturnus vulgaris*) were collected from feedlots within multiple geographic regions within the United States and European starlings within all regions tested positive for ciprofloxacin-resistant *E. coli*, but prevalence differed by region. Total number of birds on feedlots were positively associated with increased cattle fecal shedding of ciprofloxacin-resistant *E. coli*. Targeted control of invasive European starlings reduced bird numbers on feedlots by 70.4%, but decreasing populations of European starlings was not associated with corresponding reductions in bovine fecal prevalence of ciprofloxacin-resistant *E. coli*. These data provide evidence for the role of wild bird depredation in feedlots contributing to fecal shedding of ciprofloxacin-resistant *E. coli*, but a single month of European starling control in feedlots was not sufficient to impact the fecal carriage of this organism in cattle.

## Introduction

Antimicrobial resistant (AMR) bacteria constitute a significant portion of the emerging infectious disease (EID) pathogens that have been reported since 1940, and analyses of EID events suggest socioeconomic drivers (e.g., human population density, antibiotic drug use, and agricultural practices) are major determinants of their temporal and spatial patterns^1–3^. Domestication of animals appears to be a driver of EID events^4^, especially within concentrated animal feeding operations where livestock production is associated with the emergence of AMR bacteria^5–7^. Currently, there are gaps in our understanding of how AMR bacteria are maintained and moved through the food chain to human populations^8^. One potential gap in our understanding is the ecological interactions between wildlife, livestock, and people.

The presence of AMR bacteria is also an economic concern for cattle producers because AMR pathogens can contribute to increased morbidity and mortality that will increase production expenses^9,10^. Additionally, multiple antibiotics have been used as growth promotants in cattle production systems and concern over the emergence of AMR bacteria has led the Food and Drug Administration (FDA) to amend guidelines on use of antibiotics^7^. When fully implemented, these new guidelines will limit the use of medically important antibiotics for growth promotion^11^. Thus, efforts designed to reduce public health risk associated with AMR pathogens may also reduce profitability for feedlot operators.

The objective of this study was twofold: (1) assess the relationship between bird abundance and ciprofloxacin-resistant *Escherichia coli* within cattle feedlots in the United States; (2) determine the efficacy of targeted invasive species management (removal of European starlings; *Sturnus vulgaris*) as a potential pre-harvest intervention strategy to reduce cattle fecal shedding of ciprofloxacin-resistant *E. coli*. Specifically, we wanted to determine if higher total bird numbers were associated with increased cattle fecal shedding of ciprofloxacin-resistant *E. coli* and if the removal of invasive European starlings would reduce cattle fecal shedding of ciprofloxacin-resistant *E. coli*, relative to comparable reference facilities not subjected to starling control operations.

## Methods

We conducted this study from December 4, 2012 through March 12, 2013 with the cooperation of 35 feedlots. Feedlots were located within 4 regions; Eastern Colorado (n = 8), Kansas (n = 8), Texas panhandle (n = 8), Southern Iowa/Northern Missouri (n = 11). Feedlots experiencing bird damage (large foraging flocks of birds) were identified with the help of local cattlemen’s associations. Bird damaged feedlots were randomly selected from a pool of commercial facilities, within each region, that had reported severe bird damage the previous year. Comparable reference feedlots were selected within each geographical region. A total of 18 treated and 17 reference facilities were included in the analysis. All participating facilities group housed animals in pens and produced feeder cattle as their primary commodity. Dairies, calving, or non-cattle livestock facilities were not included in the study.

This experimental protocol was approved by the USDA/APHIS/Wildlife Services, National Wildlife Research Center prior to data collection (Study Director James C Carlson; NWRC Protocol number QA-1945). Starling control operations were conducted by biologists from the United States Department of Agriculture/APHIS/Wildlife Services. Starling control was conducted following agency policy as stated in USDA/APHIS/WS Directive 2.505. All methods were carried out in accordance with relevant guidelines and regulations.

Wildlife Services biologists initially established starling feeding sites within the 18 treatment feedlots using a bait preferentially selected by European starlings. Once starlings were observed to be consistently feeding on pre-bait, biologists used a 2% solution of DRC-1339 (3-chloro-p-toluidine hydrochloride) to reduce the number of depredating starlings. Technical DRC-1339 powder was mixed with water to create a 2% solution. Starling feed was soaked in the 2% solution and screen dried. The bait was applied at a concentration of 1:10 treated to untreated starling feed particles. All DRC-1339 applications were implemented consistent with directions “Compound DRC-1339 Concentrate – Feedlots”; EPA registration 56228–10.

Each feedlot was sampled twice, once before and once after starling control operations. During each sampling period we collected European starlings (n = 30) and cattle feces (n = 50) from within feedlots. Within each feedlot up to 10 pens were selected. These same pens were sampled before and after starling control operations. Within each pen we collected a minimum of five cattle fecal samples per visit. If a feedlot had fewer than 10 pens the total number of samples was distributed, as evenly as possible, among the available pens. For example, one facility housed animals in 2 large pens. Within this feedlot we collected 25 fecal samples per pen per visit. Within some feedlots fewer than 30 starlings were collected if birds could not be found.

Collection of cattle fecal samples followed methods that have been described previously^12^. Cattle fecal samples were collected from the floor of animal pens and only freshly voided fecal pats were sampled. In other words, the sample was collected from a fecal pat only after an animal was observed defecating. This procedure allowed us to standardize environmental exposure time among fecal samples and estimate herd prevalence of ciprofloxacin-resistant *E. coli* without confining animals for collection of rectal samples. Ten gram samples were scraped from the top of the fecal pat with disposable plastic spoons and stored in sterile Whirl-Paks (Nasco, Fort Atkinson, WI). We only collected fecal samples if we could reasonably determine, by visual inspection, that the sample was fresh and free of external environmental contaminants. All fecal samples were stored in coolers until they were shipped to the laboratory. Estimates of number of birds in animal pens were collected at the same time as fecal sample collection.

Number of birds on feedlots were estimated using counts of bird numbers on each pen’s floor, feed bunkers, water troughs and feed lanes in front of the sampled pen. Estimates from these four locations were summed to calculate the total number of birds within pens. We averaged the total number of birds within pens among all the sampled pens within a feedlot. This mean bird estimate was multiplied by the total number of pens within the facility to produce a facility level bird estimate.

All starlings were collected with shotguns and no birds were collected off feedlots. All starlings were collected from within the animal pens and pen lanes. Starling samples were collected opportunistically and only done when it was safe to discharge firearms in feedlots. All specimens were individually bagged in sterile Whirl-Paks and stored in coolers until shipping.

Within each facility, diagnostic samples (starlings and cattle fecal samples) were collected on the same day and samples were shipped priority overnight to testing laboratories in Iowa and Colorado. All samples were shipped, in insulated boxes packed with Ice-Brix (Polar Tech Industries, Genoa, IL), to laboratories for isolation of ciprofloxacin-resistant *E. coli*. Only samples received by the laboratories within 24 hours of the date of collection were screened for ciprofloxacin-resistant *E. coli*. European starlings were shipped to the United States Department of Agriculture, National Wildlife Research Center (NWRC) in Fort Collins, Colorado, USA. Cattle fecal samples were shipped to Ohio State University, Food Animal Health Research Program in Wooster, OH, USA.

All European starling dissections occurred at the NWRC and were conducted using published methods^13^. Starling lower gastrointestinal tracts (GI, duodenum to the cloaca) were removed and placed in a sterile Whirl-Paks. To reduce risk of cross-contamination, we saturated the starling carcass, scissors, scalpels, and lab stations with 70% ethanol before removal of each starling GI tract. Lab mats and gloves were replaced after processing each starling. The starling GI samples were macerated for 120 sec at 200 rpm using a Stomacher 80 Biomaster (Seward Laboratory Systems, Bohemia, NY) paddle blender. Fecal material from the macerated starling GI tracts was squeezed by hand to one corner of the bag and an aliquot was extracted using sterile cotton swabs, making sure to completely saturate the tip of the swab. Starling fecal material, on the saturated cotton tipped swab, was then used for direct plating onto selective media.

Starling GI and cattle fecal samples were inoculated onto MacConkey agar (HiMedia, Mumbai, India) supplemented with 1 µg/ml ciprofloxacin (Sigma-Aldrich, St. Louis, MO) using sterile cotton-tipped applicators and incubated at 37 °C for 18–24 hr. Colonies displaying typical *E. coli* morphology were transferred to 10 ml of tryptic soy broth (TSB) and incubated overnight at 37 °C for 18–24 hours. Species confirmation for starling GI samples was achieved using the API 20E system (bioMérieux, Marcy-l'Étoile, France). *E. coli* susceptibility to ciprofloxacin was confirmed using the disk diffusion method following Clinical and Laboratory Standards Institute protocols and guidelines (CLSI, 2008). Species confirmation for cattle fecal samples was conducted using lactose and indole tests. All lactose and indole positive isolates were cultured on MacConkey agar supplemented with 2 µg/ml ciprofloxacin. Colonies growing on the agar were isolated and tested for both gyrA and parC chromosomal mutations by PCR using previously reported primers^14^. PCR products were bi-directionally Sanger sequenced and the resulting data were aligned to the corresponding reference gene sequences available in NCBI Genbank (*gyrA* gene ID: 946614; *parC* gene ID: 947499). The *gyrA* and *parC* sequences were screened for combinations of chromosomal mutations expected to confer fluoroquinolone resistance^15,16^ and if they were detected the *E. coli* isolate was classified as ciprofloxacin-resistant.

We tested efficacy of DRC-1339 as a control tool for invasive birds on feedlots using a Poisson model of count data in PROC GLIMMIX in SAS version 9.2 (SAS Institute, Cary, NC). The response variable was the estimated number of birds on feedlots. Fixed effects included treatment status (starling controlled feedlot/reference feedlot), sampling period (before/after starling control) and the interaction between treatment status and sampling period. Feedlots nested within treatment status were included as a random effect.

Separate mixed effects logistic regression models were created to test the association between total bird number and ciprofloxacin-resistant *E. coli* fecal shedding by cattle and to test the efficacy of starling control as a pre-harvest intervention strategy to reduce ciprofloxacin-resistant *E. coli* fecal shedding by cattle. Models were constructed using PROC GLIMMIX in SAS version 9.2. Both models, were fitted using a binomial distribution and the response variable was the number of positive ciprofloxacin-resistant *E. coli* samples divided by the total number of samples collected per pen. Model parameters were estimated using the maximum likelihood method and degrees of freedom were estimated using the between within option. Within both models, feedlots nested within treatment status, pens nested within feedlots, and the sampling period by feedlot interaction were all included as random effects.

To test for an association between total bird numbers and cattle fecal shedding of ciprofloxacin-resistant *E. coli*, we included region and the estimated number of birds on feedlots as fixed effects. To test the efficacy of starling control as a pre-harvest intervention strategy to reduce cattle fecal shedding of ciprofloxacin-resistant *E. coli*, we included region, treatment status (starling controlled feedlot/reference feedlot), sampling period (before/after starling control operations), and the interaction between treatment status and sampling period as fixed effects.

Additional explanatory variables for ciprofloxacin-resistant *E. coli* in feedlots were assessed in univariable analyses using PROC GLIMMIX in SAS version 9.2. The model was fitted using a binomial distribution and the response variable was the number of positive ciprofloxacin-resistant *E. coli* samples divided by the total number of samples collected per pen. Model parameters were estimated using the maximum likelihood method and degrees of freedom were estimated using the between within option. Feedlots nested within treatment status, pens nested within feedlots, and the sampling period by feedlot interaction were all included as random effects.

The additional explanatory variables were assessed to identify any potential wild bird, facility management, or environmental variables that may potentially be associated with cattle fecal shedding of ciprofloxacin-resistant *E. coli* in feedlots. The explanatory variables assessed in the analyses were selected because they have been identified as or suspected of contributing to bacterial contamination in feedlots^13,17–20^. The variables assessed in these analyses occurred at two spatial scales (feedlots and pens within feedlots). The variables include birds (birds in feed bunkers, birds on water troughs, total number of birds in pens), cattle stocking (herd size, number of cattle within pen), environmental factors (temperature, time, and sampling period), and feedlot management factors (water troughs split pens, recycled water used in water troughs, cattle days in pen, cattle days on finishing ration, entry weight, exit weight and weight gained by cattle).

Most variables assessed within the univariable analyses are intuitively obvious, but some variables may need additional clarification. For example, weight gain was calculated by subtracting the pen averaged entry weight from the pen averaged exit weight data. Water troughs accessed by multiple pens identifies split-pen watering troughs that allow cattle from adjoining pens to drink from the same trough. Recycled water identifies facilities that recirculate the water provided to cattle within troughs. Total number of birds per pen reflects the sum of the estimates of birds from water troughs, pen floor, feed bunkers and pen lanes for each sampled pen.

A total of 15 additional univariable models were analyzed (*m* = 15). Because multiple tests were being conducted, we decided to control for false discoveries using the Benjamini Hochberg procedure^21^. For all univariable analyses the false discovery rate was set at α = 0.05. Models were ranked by p-values from smallest (1) to largest (*m*). Cutoff values for rejection of null hypotheses were calculated as (rank/*m*)*α. Reported odd ratios and their 95% confidence intervals were not adjusted for multiple testing.

## Results

**Figure 1:**
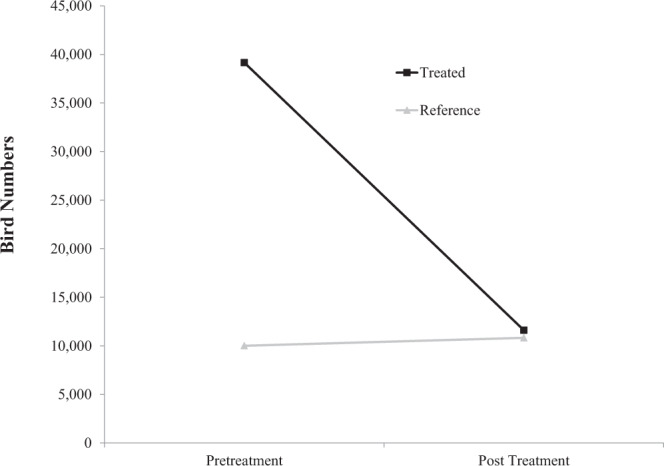
Mean number of European starlings (*Sturnus vulgaris*) observed on starling controlled (treated) and non-starling controlled (reference) feedlots before and after DRC-1339 applications within Kansas, Texas, Colorado, Missouri and Iowa, USA in 2013.

Targeted control of invasive European starlings using DRC-1339 was effective at reducing bird numbers on feedlots. Total number of birds on treatment facilities relative to the reference facilities not subjected to control operations decreased following DRC-1339 control operations (F_1_,_33_ = 95,598, P = < 0.0001). Bird count data suggests targeted starling control operations reduced bird numbers by 70.4% on feedlots following DRC-1339 applications (Fig. 1).

**Figure 2:**
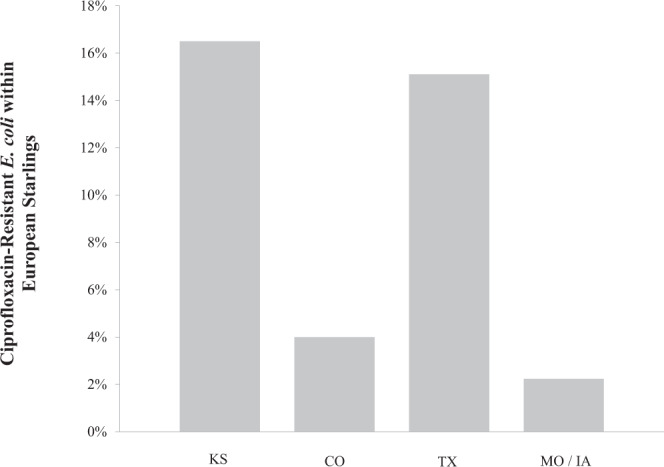
Percentage of European starling gastrointestinal tract samples that tested positive for ciprofloxacin-resistant *Escherichia coli*. Birds were collected from feedlots within Texas, Colorado, Kansas, Missouri and Iowa between December 2012 and April 2013.

A total of 1,477 European starling specimens were collected for laboratory analysis. A total of 10.2% of starling GI tracts tested positive for ciprofloxacin-resistant *E. coli* and the probability of detection within starling GI tract samples appears to differ by geographical region (Fig. 2).

**Figure 3:**
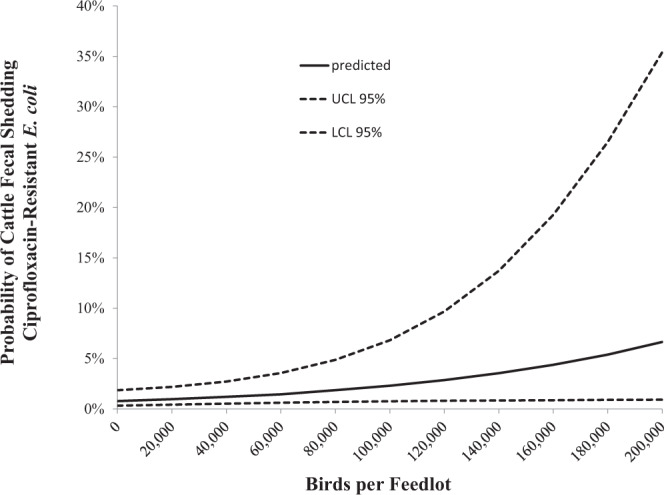
Predicted probabilities and 95% confidence intervals of a fecal pat testing positive for ciprofloxacin-resistant *Escherichia coli* as a function of bird numbers observed on feedlots within Texas, Colorado, Kansas, Missouri and Iowa between December 2012 and April 2013.

The odds of cattle shedding ciprofloxacin-resistant *E. coli* significantly varied with region (F_3_,_286_ = 5.69, P = 0.0009) and the number of birds on feedlots (F_1_,_286_ = 4.46, P = 0.0355, Table 1). The probability of detecting ciprofloxacin-resistant *E. coli* in cattle fecal samples increased as bird numbers increased on feedlots (odds ratio per 100 birds = 1.001, 95% CI = 1.000, 1.002) and effectively doubled when 65,000 birds were observed foraging on feedlots (odds ratio = 2.003, 95% CI = 1.048, 3.827, Fig. 3).

**Table 1:** Odd ratios from multivariable mixed-effects logistic regression model used to test the association between total bird numbers and cattle fecal shedding of ciprofloxacin-resistant *Escherichia coli*.

Model Variables	OR	OR 95% CI	P-value
Bird Numbers	1.001^a^	1.000–1.002	0.0356
**Region**			0.0009
Texas	Reference	Reference	
Iowa/Missouri	0.068	0.016–0.292	
Colorado	0.077	0.017–0.346	
Kansas	0.152	0.033–0.704	
Variance Components	Var(SE)		
Site(Treatment)	0.3186(0.7266)		
Pen(Site)	1.3587(0.3878)		
Period*Site(Treatment)	2.3185(1.1164)		

^a^Odds ratio report the odds of per 100 birds observed on feedlots.

**Figure 4:**
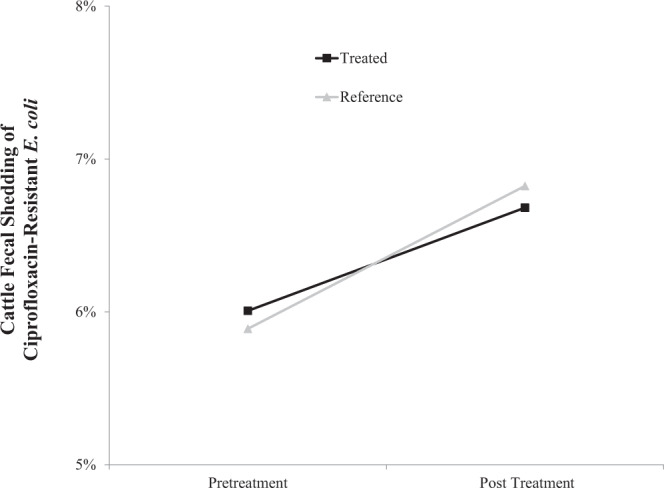
Probability of detecting ciprofloxacin-resistant *Escherichia coli* in cattle feces on starling controlled (treated) and non-controlled (reference) feedlots before and after DRC-1339 applications within Texas, Colorado, Kansas, Missouri and Iowa between December 2012 and April 2013.

Targeted control of invasive European starlings was not an effective pre-harvest intervention strategy to reduce cattle fecal shedding of ciprofloxacin-resistant *E. coli* (F_1_,_33_ = 0.60, P = 0.4454, Table 2). Based on LS-Means estimates of ciprofloxacin-resistant cattle fecal samples there does not appear to be any reduction in cattle fecal shedding of ciprofloxacin-resistant *E. coli* within starling controlled feedlots relative to reference feedlots (Fig. 4).

**Table 2:** Odds ratios from multivariable mixed-effects logistic regression model used to test the efficacy of targeted control of invasive bird species as a pre-harvest intervention strategy to control ciprofloxacin-resistant *Escherichia coli* cattle fecal shedding.

Model Variables	OR	OR 95% CI	P-value
Sampling Period			
Post versus pre-treatment sampling	1.410	0.550–3.616	0.4630
**Treatment status**			
Reference versus treatment feedlots	0.883	0.268–2.911	0.8323
**Period x Treatment**			
Period x Treatment Interaction			0.4454
Variance Components	Var(SE)		
Site(Treatment)	0.8339(0.8252)		
Pen(Site)	1.3055(0.3716)		
Period*Site(Treatment)	2.3325(1.1603)

The analysis of the 15 univariable models of potential explanatory variables for cattle fecal shedding of ciprofloxacin-resistant *E. coli* did not reveal any statistically significant associations after Benjamini Hochberg adjustments were made (Table 3).

**Table 3:** Odds ratios, confidence intervals for odds ratios, P-values, P-value rank, and calculated Benjamini Hochberg cutoff values for significance of univariable models examining the odds of a fecal pat from cattle in commercial feedlots testing positive for ciprofloxacin-resistant *Escherichia coli* and independent variables related to starling population, pen, and feedlot characteristics.

Variable^a^	OR^b^	OR 95% CI^c^	P-value	Rank^d^	(rank/m)*α^e^
Days on Finishing Ration	1.445	1.032–2.023	0.0320	1	0.003
Cattle Entry Weight	0.998	0.996–1.000	0.0645	2	0.007
Number of Cows per Pen	0.996	0.992–1.001	0.1436	3	0.010
Cattle Exit Weight	0.998	0.995–1.001	0.2022	4	0.013
Cattle Days in Pen	1.003	0.997–1.009	0.3261	5	0.017
Number of Birds in Water Troughs	1.017	0.980–1.056	0.3726	6	0.020
Number of Cattle in Feedlot	1.010	0.983–1.037	0.4670	7	0.023
Sampling Period	1.417	0.538–3.735	0.4692	8	0.027
Weight Gained by Cattle	1.001	0.999–1.003	0.4829	9	0.030
Troughs Recycle Water	1.334	0.090–19.792	0.8289	10	0.033
Water Troughs Split Pens	0.575	0.086–3.837	0.8396	11	0.036
Time of Day Sample Collected	1.000	0.997–1.003	0.8571	12	0.040
Daily High Temperature	0.996	0.994–1.050	0.8822	13	0.043
Number of Birds in Pen	1.000	0.999–1.001	0.9198	14	0.047
Number of Birds in Feed	1.000	0.997–1.002	0.9210	15	0.050

^a^Analyses of variables potentially associated with ciprofloxacin-resistant *Escherichia coli* contamination of cattle feed samples

^b^Odds ratios for variables potentially associated with ciprofloxacin-resistant *Escherichia coli* fecal shedding by cattle.

^c^95% confidence intervals for odds ratios of potential explanatory variables associated with ciprofloxacin-resistant *Escherichia coli* fecal shedding by cattle.

^d^Rank order of p-values from analyses of potential explanatory variables associated with ciprofloxacin-resistant *Escherichia coli* fecal shedding by cattle.

^e^Benjamini Hochberg cutoff values for rejection of null hypotheses. Based on these cutoff values, we failed to reject null hypotheses for all univariable analyses assessing ciprofloxacin-resistant *Escherichia coli* fecal shedding by cattle.

## Discussion

Wildlife incursions into animal agricultural operations have long been suspected as sources for diseases of concern to veterinary and human health^22–24^. For example, indistinguishable AMR *S. enterica* isolates were recovered from starlings, cattle, and the feed and water sources they share^13,20^. Additionally, cattle fecal pats showing reduced susceptibility to cefotaxime and ciprofloxacin were spatially correlated to starling night roosts in Ohio^25^. Proximity of starling night roosts was also shown to be spatially correlated with increased *E. coli* O157:H7 cattle fecal shedding in dairies^26^. These data are important because they provide indirect evidence that bird-livestock interactions may contribute to rates of cattle fecal shedding of *E. coli* 0157:H7 as well as *S. enterica* and *E. coli* with reduced susceptibilities to multiple antibiotics, including ciprofloxacin and cefotaxime. The data we present in this manuscript are the first to provide direct evidence to support the hypothesis that large foraging flocks of birds can contribute to increased cattle fecal shedding of ciprofloxacin-resistant *E. coli*.

Starling control operations reduced bird numbers on our treatment feedlots by an average of 70.4%. Yet, the time between pre treatment and post treatment sampling did not result in any significant change in cattle fecal shedding of ciprofloxacin-resistant *E. coli*. One would intuitively assume that significant reductions in bird numbers on feedlots should translate to cattle harboring fewer organisms with reduced susceptibilities to ciprofloxacin. It is unclear why we did not see a significant reduction in the amount of ciprofloxacin-resistant *E. coli* isolated from cattle fecal pats, while seeing a positive correlation between bird numbers and cattle fecal shedding of ciprofloxacin-resistant *E. coli*. We suspect the time between starling control operations and post-treatment sampling may not have been long enough for these management actions to produce meaningful results. If so, starling control may have to occur year round or the moment starlings arrive on feedlots in the fall for it to be effective at reducing the amplification and spread of AMR organisms in animal agricultural operations.

It is important to note that other studies have shown that bird control was not an effective pre-harvest intervention strategy for reducing cattle fecal shedding of bacteria of concern to public health. Starling numbers were one of the strongest predictors for *S. enterica* contamination of cattle feed and water supplies, but starling numbers were not shown to be a good predictor for herd level prevalence of *S. enterica*^27^. Controlling starlings was associated with reduced *S. enterica* loads within cattle feed and water supplies, but starling control was not effective at reducing cattle fecal shedding of *S. enterica* over the time period of the study. Additionally, starling control programs were not an effective intervention strategy to reduce the overall prevalence of *Campylobacter jejuni* within feedlot cattle despite starlings harboring diverse *C. jejuni* strains including hypervirulent clone SA^28^. The totality of this information is discouraging. Bird numbers and bird depredation in feedlots and dairies is associated with higher herd level prevalence for multiple zoonotic and AMR organisms, but temporarily or transiently reducing bird numbers, after they have become established in animal agricultural operations, does not translate to quick reductions of herd level prevalence of those same organisms. In other words, once AMR organisms have been introduced by starlings, they may persist within cattle herds for considerable periods of time.

After population control programs were completed, approximately 30% of the pretreatment birds remained on feedlots. It is conceivable that the microbiological impact of birds is not additive and that only a few birds, moving between feedlots and dairies, are necessary for maintenance and amplification of ciprofloxacin-resistant *E. coli* in concentrated animal feeding operations. Additional studies are needed to better assess interactions between birds, cattle and the occurrence of AMR *E. coli*. For example, there is very little information related to antibiotic usage in agriculture, wildlife interactions and selective pressure on the maintenance of ciprofloxacin-resistance *E. coli* in livestock. It is conceivable that wildlife are contributing to these problems in complex and unforeseen ways. To adequately address public and environmental health concerns created through wildlife-livestock interactions we need to understand the specific risks created by wildlife so we can develop targeted and cost effective management strategies.

Bird-livestock interactions in animal agricultural operations may create an ecologically important link for the spread of ciprofloxacin-resistant *E. coli* to human populations. Synanthropic birds, especially European starlings, use feedlots in winter for food resources. Starlings typically quit using feedlots in spring when insects become abundant^29^. During the spring and summer, starlings are commonly found breeding in suburban and urban environments^30,31^. The ecological interactions of starlings suggest they could potentially move ciprofloxacin-resistant *E. coli* and other AMR organisms to environments dominated by people; human-bird transfer of *E. coli* has been documented before^32^. Birds seem to act as transporters, or as reservoirs, of resistant bacteria and could therefore have an important epidemiological role in the dissemination of resistance^33^. Thus, because of the unique ecology of invasive starlings in North America, they are a high risk species for the environmental dissemination of AMR organisms to environments and locations of concern to people.

## Data Availability

All raw data are archived at the National Wildlife Research Center (Study Director James C Carlson; NWRC Protocol number QA-1945) and are publicly available. Names and addresses of cooperating feedlots have been redacted from the raw data. All facilities were referenced by an alpha-numeric code and names and addresses of cooperating facilities will not be provided upon request as per the cooperator agreement established prior to data collection.
